# Innovative Composite Resin Restoration Techniques in Posterior Permanent Teeth of Young Patients: Presentation of Two Clinical Cases

**DOI:** 10.1155/crid/6675239

**Published:** 2025-11-09

**Authors:** Konstantina Chatzidimitriou, Victoria Katechi, Kyriaki Seremidi

**Affiliations:** ^1^Department of Preventive & Community Dentistry, School of Dentistry, National and Kapodistrian University of Athens, Athens, Greece; ^2^Department of Paediatric Dentistry, School of Dentistry, National and Kapodistrian University of Athens, Athens, Greece

**Keywords:** direct composite restorations, injection molding technique, stamp technique

## Abstract

**Introduction:**

This case report highlights the clinical application of stamp and injection molding techniques for the restoration of young permanent teeth, as efficient, esthetic, and less invasive alternatives to conventional restorative approaches. However, each has distinct limitations that should be taken into consideration for optimal clinical outcomes.

**Report:**

The first case was an anxious 14-year-old girl, who presented to the Postgraduate Clinic of Paediatric Dentistry Department (NKUA), for the restoration of a right upper first permanent molar. Intraoral examination confirmed an extensive occlusal carious lesion involving the distobuccal cusp. Given the need for direct esthetic and functional restoration, the injection molding technique was selected as the most appropriate treatment approach ensuring precise anatomical replication and seamless restoration with the least clinical time and effort, reducing the stress of the patient. The second case was a 9-year-old girl, also examined at the clinic with carious lesions on the occlusal surfaces of the first permanent mandibular molars, but without extensive tissue loss. Given the nature of the lesions, the stamp technique was chosen as the preferred method of restoration, allowing for quick and efficient restoration by replicating the tooth's original morphology. Both techniques provided functional and esthetic results, proving their effectiveness in pediatric restorative dentistry while minimizing chairside time and preserving natural tooth structure.

**Conclusion:**

Stamp and injection molding techniques offer promising alternative solutions for direct composite restorations of posterior permanent teeth in young patients. Their ability to provide predictable, esthetic, and efficient restorations underscores their clinical significance. However, careful case selection, meticulous application, and periodic recall examinations remain essential to optimize their outcomes and ensure long-term success.

## 1. Introduction

Patients' expectations, that nowadays play a critical role in the treatment planning and the outcome of the interventions, focus not only on esthetic excellence but also on minimizing time spent in the dental chair, reducing discomfort, and ensuring cost-effectiveness. This in combination with the evolution of dental materials and the deeper understanding of their physical and mechanical properties in recent years has enabled clinicians to integrate innovative techniques that successfully balance these factors while also providing high-quality restorations. These techniques are, therefore, gaining increasing attention and in some cases are preferable as a treatment method compared to conventional techniques [1].

One such technique is the so-called “stamp technique,” a technique that directly replicates the natural/physiological anatomy of the tooth and transfers it directly to the final restoration. It is particularly applicable in cases where the tooth damage is minimal and has not altered the occlusal morphology. It is more suitable for Class I and II lesions, although reports support its effectiveness in eroded surfaces too [2, 3]. Initially, a kind of “stamp” is made through a copy of the occlusal surface using either a silicone key or a flowable composite resin in which a microbrush or any fine-tipped instrument is dipped. This replica of the occlusal surface is applied in the final layer of the composite resin before it is polymerized to reproduce the pit and fissure anatomy [4, 5].

The primary advantage of this technique is the predictability of the result, allowing for accurate reproduction of the tooth's morphology with relative ease and minimal technical complexity. The restoration is completed in a short time with minimal material waste, yielding satisfactory results. Studies and clinical cases indicate that restorations completed using this technique exhibit good longevity and excellent esthetic outcomes [6]. Additionally, the stamp technique is particularly beneficial for cases with extensive lesions, where occlusal reference points are missing, as it facilitates anatomical reconstruction while reducing potential postrestorative sensitivity due to occlusal discrepancies.

However, one limitation is the lack of marginal integrity, as microfractures and chipping at the restoration's margins may develop over time, leading to microgaps. The use of flowable composite resin can also result in material overflow, requiring meticulous finishing and polishing of the restoration. Furthermore, while the technique effectively replicates overall tooth anatomy, it may not perfectly reproduce finer occlusal details such as deep grooves and fissures [7].

Another emerging technique in restorative dentistry is the injection molding technique, which leverages the viscosity and superior esthetic properties of flowable composite resins [8–10]. This method is particularly useful for restoring posterior lesions, as well as cases involving teeth with developmental anomalies, poor anatomy, or esthetic deficiencies requiring correction of shape, size, or appearance. Additionally, it has been used as a temporary solution in cases of amelogenesis imperfecta, helping to prevent further enamel loss and improve sensitivity before the patient's growth is complete and a more definite restoration can be performed [11, 12].

The injection molding technique requires an impression and a wax-up preparation, followed by the fabrication of a silicone guide or transparent omnivac matrix. Traditionally, wax templates were used; however, advancements in 3D printing technology now allow for the creation of digital models, significantly improving precision by eliminating human errors [13]. After ensuring proper isolation of adjacent teeth with Teflon strips, the prepared mold is positioned in the teeth and is filled with flowable bulk-fill composite resin through a small hole that has been made. This technique provides highly esthetic, predictable, and functional results, although its main disadvantage is its “indirect” nature as it requires two visits increasing the chair time for the patient. Being a less invasive approach, it aligns with modern dentistry principles, offering an efficient, cost-effective, and time-saving solution that satisfies both parents and young patients. Despite its advantages, the injection molding technique does have limitations. The most reported concerns include discoloration, pigment absorption, surface roughness, and marginal chipping, which may necessitate periodic follow-ups and refinements. Additionally, some studies highlight potential issues with bond strength and durability, particularly regarding long-term marginal adaptation [14–16].

However, the search for information in the literature about the efficacy and acceptability of these techniques returns scarce results due to their relative newness in restorative dentistry. Therefore, the present case report was aimed at describing in detail the use of both injection molding and stamp techniques for the restoration of permanent posterior teeth in two young patients.

## 2. Case 1

**Figure 1:**
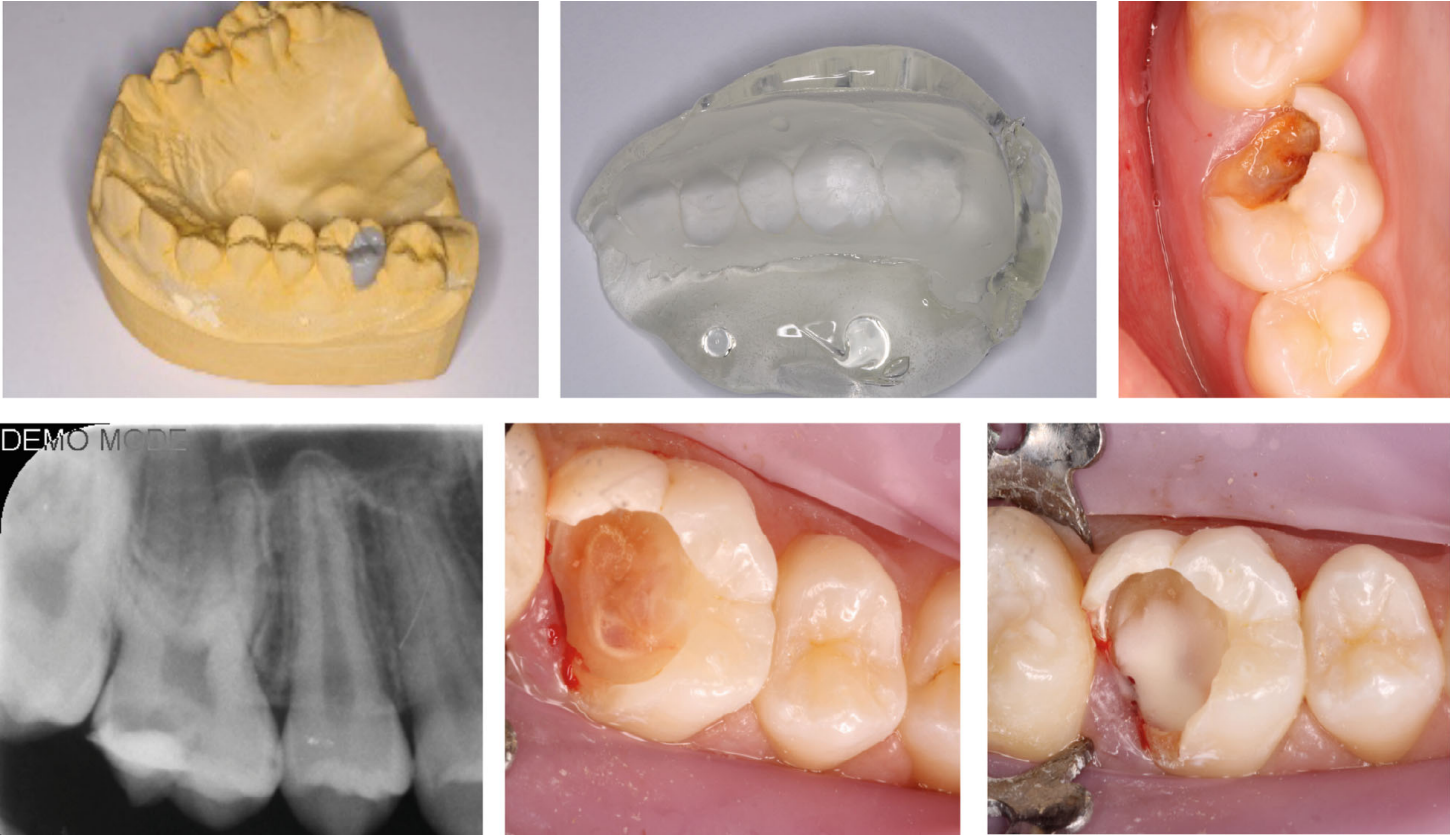
Intraoral clinical and radiographic appearance confirming an extensive occlusal carious lesion in the right upper first permanent molar involving the distobuccal cusp, in close proximity to the pulp. Preparation of a wax-up model by a specialized dental technician and fabrication of a silicone matrix.

A 14-year-old Caucasian girl, with a noncontributory medical history, presented to the Paediatric Dentistry Department (NKUA) for restoration of her right upper first permanent molar [16]. The patient reported no signs or symptoms of pain or sensitivity. The intraoral examination confirmed an extensive occlusal carious lesion involving the distobuccal cusp (ICDAS 6), without tenderness to percussion or sensitivity upon palpation radiographically observed to be in close proximity to the pulp (Figure 1). Based on the ICDAS criteria, the lesion was classified as ICDAS Code 6, representing extensive cavitation with visible dentin involving at least half of the tooth surface.

During examination, the patient was anxious and fearful due to a previous bad experience (Frankl 2). Despite crying and her incessant talk, she showed no pronounced refusal and signs of willingness to cooperate with the presence of her mother. This justified the choice of the injection molding technique as the most appropriate treatment approach to decrease the time required to achieve a functional and esthetically acceptable result while giving the patient more time to familiarize herself with the dental environment.

During the first appointment and consultation, nonpharmacological behavior management techniques such as tell–show–do, positive reinforcement, and clear explanations were employed to facilitate cooperation. At the same visit, an impression was taken, and the shade selection was carried out using a VITA classical shade guide (VITA Zahnfabrik, Bad Säckingen, Germany) under natural daylight conditions. In between appointments, a wax-up model was prepared by a specialized dental technician, and a silicone matrix was then fabricated using a transparent addition silicone material (Exaclear, GC Corp., Tokyo, Japan) to which two designated openings—one for injecting the flowable resin and another for material overflow—were made.

**Figure 2:**
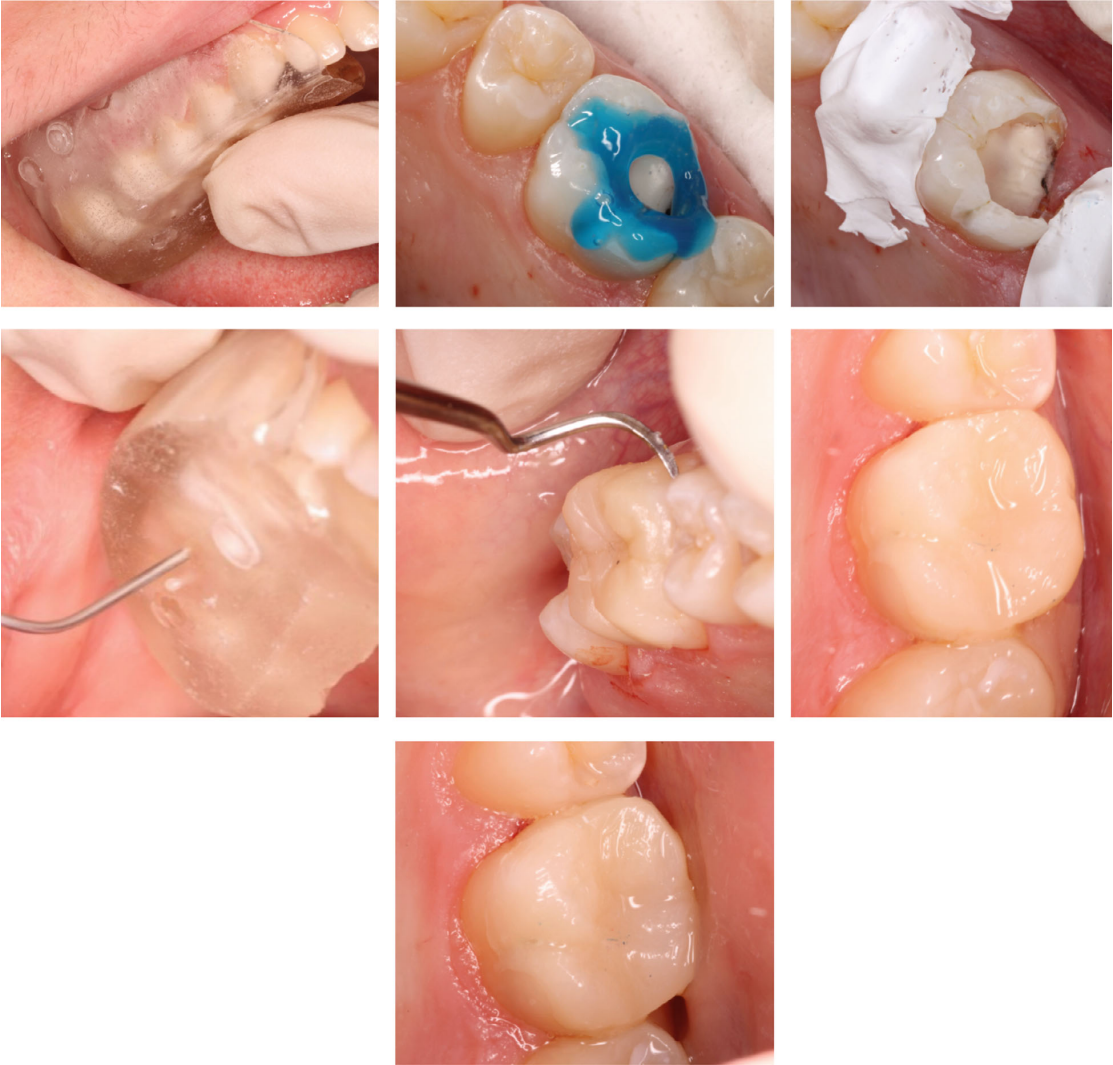
Complete caries removal was performed followed by the etching and bonding protocol. The silicone matrix was placed and the flowable resin was injected through the designated opening. After an initial photopolymerization, the matrix was carefully removed, and excess material was trimmed. The restoration was then fully polymerized and final adjustments were made. Six months posttreatment, the restoration was considered clinically acceptable, with excellent marginal integrity, no discoloration, secondary caries formation, or gross fracture.

At the second appointment, using the same basic behavioral management techniques, computer-assisted electronic anesthesia was delivered (4% articaine with 1:100,000 epinephrine [Dentsply Sirona, York, PA, United States]) to allow painless complete caries removal under rubber dam isolation. The rubber dam was removed to allow correct placement of the silicone matrix, and isolation was maintained using cotton rolls and Teflon strips to protect adjacent teeth and ensure a dry operating field. The etching protocol included the application of 37% phosphoric acid gel (Scotchbond Etchant, 3M ESPE, St. Paul, MN, United States) then rinse and gently air-drying to maintain dentin moisture. A one-component light-cured universal adhesive (G-Premio Bond, GC Corp., Tokyo, Japan) was applied for 20 s, gently air-thinned for 5 s, and light-cured for 20 s. Then, the bulk-fill flowable composite resin (G-aenial Universal Injectable, GC Corp., Tokyo, Japan) was injected through the designated opening of the matrix (Figure 2). After an initial photopolymerization of 10 s, using a LED-curing unit (Bluephase N, Ivoclar Vivadent, Schaan, Liechtenstein) with an output intensity of approximately 1.200 mW/cm^2^ at a wavelength range of 430–490 nm, the matrix was carefully removed, and excess material was trimmed using a #12 scalpel blade. The restoration was then fully polymerized for an additional 30 s under the same conditions at a distance of ~1 mm from the surface to ensure adequate depth of cure. Final adjustments included contouring with fine-grit diamond burs and Sof-Lex discs (3M ESPE, St. Paul, MN, United States) in descending order of abrasiveness. Polishing was performed with rubber polishing cups, and a final gloss was achieved using diamond polishing paste (Prisma Gloss, Dentsply Sirona, York, PA, United States). Occlusal contacts were checked and adjusted as necessary to ensure a natural and seamless restoration.

Six months posttreatment restoration was considered clinically acceptable, with excellent marginal integrity, no discoloration, secondary caries formation, or gross fracture. The patient did not report any symptoms and was fully functional and happy with the outcome.

## 3. Case 2

A 9-year-old Caucasian girl, with a noncontributory medical history, was examined at the Pediatric Dentistry Department (NKUA). Clinical and radiographic examinations revealed carious lesions on the occlusal surface of the first permanent mandibular molar (46), but without extensive tissue loss and no signs or symptoms of pain or sensitivity reported by the patient. Given the nature of the lesion, the stamp technique was chosen as the preferred method of restoration to reduce extensive and unnecessary tissue removal and also the chair time required to decrease the patient's discomfort.

**Figure 3:**
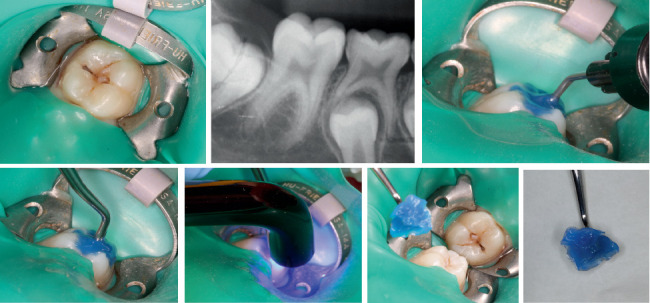
Clinical and radiographic examination revealed a carious lesion on the occlusal surface of the first permanent mandibular molar. Following rubber dam isolation and without any preparation, a flowable resin was placed on the occlusal surface, and a thin-tipped instrument was dipped into it before being polymerized.

**Figure 4:**
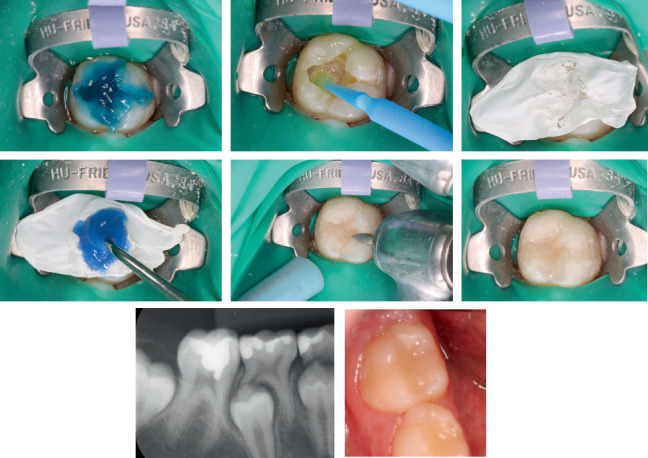
Following standard protocols, etching, bonding, and resin layering proceeded up to the final stages. At this point, the preformed stamp was applied over the composite, with a Teflon separator placed between the layers to ensure material separation. The restoration was then polymerized, and final adjustments were made. Clinical and radiographic examination 8 months posttreatment showed that occlusal integrity and morphology have been maintained without secondary caries formation and any other pathologic findings.

Treatment was performed in one appointment, under conventional inferior alveolar nerve block anesthesia (4% articaine with 1:100.000 epinephrine [Dentsply Sirona, York, PA, United States]) and rubber dam isolation. Before caries removal and without any initial mechanical or chemical preparation, the recording of the anatomical contour of the tooth was achieved by placing a flowable resin with a thin-tipped instrument dipped into it on the occlusal surface (Figure 3). Upon caries removal, the pulp was exposed, and a partial pulpotomy was performed using NeoPutty bioceramic material (Avalon Biomed, Bradenton, FL, United States) before final restoration. Following standard protocols, etching with 37% phosphoric acid gel (Scotchbond Etchant, 3M ESPE, St. Paul, MN, United States) was performed, and then the cavity was rinsed and gently air-dried, leaving dentin moist. A universal adhesive system (Scotchbond Universal Adhesive, 3M ESPE, St. Paul, MN, United States) was then applied actively for 20 s, air-thinned for 5 s, and light-cured for 20 s. For the restoration, a nanohybrid composite resin (Filtek Z250 XT, 3M ESPE, St. Paul, MN, United States; Shade A2) was placed in incremental layers of ≤ 2 mm thickness to reduce polymerization shrinkage and ensure adequate curing. Each increment was light-cured for 20 s using an LED-curing unit (Bluephase N, Ivoclar Vivadent, Schaan, Liechtenstein; 1200 mW/cm^2^, 430–490 nm). In the final stage, the preformed stamp was applied over the last uncured composite increment, with a Teflon separator placed between the layers to ensure material separation (Figure 4). The restoration was then polymerized, and final adjustments were made, including contouring with fine-grit diamond burs and finishing with Sof-Lex discs (3M ESPE, St. Paul, MN, United States) in decreasing abrasiveness and polishing with rubber cups (Enhance, Dentsply Sirona, York, PA, United States). A final high-gloss polish was achieved using diamond polishing paste (Prisma Gloss, Dentsply Sirona, York, PA, United States). Occlusal contacts were checked and adjusted to ensure a highly esthetic and functional restoration that accurately replicated the original tooth morphology.

Eight months posttreatment occlusal integrity and morphology have been maintained without secondary caries formation.

## 4. Discussion

The present case report highlighted the clinical application and effectiveness of two alternative techniques, the stamp and injection molding techniques, in the restoration of posterior permanent teeth in young patients. Both presented viable alternatives to conventional direct composite resin restorations, providing advantages regarding efficiency, esthetic outcomes, and ease of use. However, each method has limitations that must be carefully considered in clinical practice.

Direct composite restorations have proven effective for small cavities [17], but in cases with extended carious lesions or endodontically treated teeth, free-hand restoration of anatomical morphology can be challenging, especially in young patients with difficulties in cooperation [18–21]. Better outcomes could be achieved by planning and simulating the appropriate tooth morphology before treatment and then accurately transferring it to the treated tooth. Therefore, from this clinical point of view both techniques could be beneficial [1].

In the first case, the molding injection technique was selected as the most appropriate compared to indirect restorations due to its shorter chair time, lower cost, and more predictable outcomes [17, 22]. Although two sittings and additional laboratory steps were required, the actual chairside clinical time was reduced compared to a conventional direct build-up. This was particularly beneficial for our anxious patient, as the matrix-guided application significantly eliminated incremental sculpting and occlusal anatomy reproduction making the overall clinical experience less stressful and more predictable. A flowable composite was used for the final restoration as these materials are preferable to conventional ones due to their consistency [17]. They are capable of adapting to the shape of the transparent silicone index and, consequently, to the diagnostic wax-up, without requiring external pressure. New flowable resin composites with a greater filler content (69 wt%) have improved mechanical properties, strength, wear resistance, and transparency [23–25]. New resins with higher load values (61%–71% by weight) provide better material adaptation to the cavity walls as well as to the posterior walls, with fewer failures compared to conventional ones [26]. Additionally, according to recent meta-analyses, flowable and conventional composites do not differ significantly in any of the clinical outcomes assessed [10, 24]. However, the literature is still quite limited, and its long-term application requires careful consideration [14]. This explains why recent case reports have utilized this material in conjunction with the “injectable resin composite technique” [13].

Comparing injectable resin composites to traditional restorations, however, may raise questions about their durability and mechanical properties [27]. In previous studies where conventional composite resins were used, the application of external pressure (to precisely replicate anatomy) was required, and also, the cutting of the index into segments for each tooth was necessary, both of which impair stability and accuracy [28, 29]. According to a recent study [30] on the longevity of restorations as well as the selection and use of restorative materials alone, it was reported that the success of restorations depends on a number of patient-related risk factors, including age, parafunctional habits, and the size of the restoration. This is because the more dental structure replaced by a polymeric composite, the more mechanical challenges the restoration will face.

An important consideration in the injection molding technique is the potential for polymerization shrinkage, which may cause stresses at the tooth–restoration interface, leading to marginal gap formation, postoperative sensitivity, or reduced bond durability. However, as already mentioned, recent developments in high-viscosity and highly filled flowable bulk-fill composites seem to reduce shrinkage stress while maintaining adequate depth of cure [10, 23, 24, 27]. In addition, the use of a transparent silicone matrix and light curing across different surfaces can help improve polymerization efficiency and minimize stress development. Nevertheless, careful and consistent restoration monitoring is still essential to identify any issues, including marginal discoloration or chipping [22], and to properly and timely deal with them, increasing its longevity [31]. It is worth mentioning that in the literature, there are no clinical studies that focus on the long-term follow-up of this technique, but rather only case reports, mainly reporting on the restoration of anterior teeth. Future research efforts, such as randomized controlled trials with larger sample sizes, are essential to validate these findings and evaluate long-term performance and patient satisfaction.

In the second case, the stamp technique was chosen as the most appropriate treatment approach for the restoration of a mandibular permanent molar without extensive hard tissue loss. It was initially chosen given its reduced chair-side time and ease of use given the young age of the patient. The main advantage of this method is that it uses the least amount of material possible to restore the right form, function, and esthetic structure of the tooth. Because of the immediate cusp fossa relation, it also requires minimal time to finish and polish, making it ideal especially for young patients [2]. Furthermore, the final restoration has a far lower degree of porosity due to the pressure that the stamped matrix applies to the composite, which lowers the amount of microbubbles produced and keeps oxygen from interfering with the polymerization of the final composite layer [4]. The long-term performance of composites has been shown to be significantly predicted by these characteristics [32].

Even though a stamp can be made from a variety of materials, the flowable resin stamp is the most traditional and commonly used technique, and it was also applied in the second case of the present report. On the one hand, flowable resin has a higher flowability and can more effectively enter pits and fissures than silicone materials, guaranteeing that the occlusal surface's shape can be precisely reproduced. On the other hand, because flowable resin is rigid, it may be pressed against the tooth surface without deforming, as may occur for a silicone stamp due to its elasticity [33]. Other materials, including transparent silicone-based ones, might also be used to replicate the enamel surface, but they may not be easily accessible in a general dental office and might be retained in deep fissures and pits, tearing apart when removed from the surface [2, 34].

However, the vulnerability to stamp breakage and the flowable composite's cost are some essential drawbacks of utilizing a flowable resin stamp. As shown in our case, Teflon was also used in the stamp technique. With the use of a Teflon sheet, issues with composite polymerization are addressed, preventing oxygen inhibition and ensuring complete curing without a sticky layer. This method promotes a smoother surface and reduces the possibility of material shrinkage or partial polymerization, enhancing durability and esthetic outcomes [35].

Currently, there are many published case reports on the direct restoration of posterior teeth using the stamp technique, but clinical studies are very scarce [7]. The existing literature has demonstrated that the restoration performed with the stamp technique was clinically excellent or good in terms of functional and esthetic properties, while its clinical evaluation has shown good results for as long as 40 months posttreatment [2, 7, 34].

From a clinical perspective, both methods are consistent with the principles of less invasive dentistry since as much of the tooth structure as possible is preserved while producing restorations that are both functional and esthetic. In pediatric and adolescent patients, where chairside effectiveness, patient cooperation, and long-term results are critical to treatment success, they also offer an efficient way to manage restorative cases. The lesion extent, the preservation of the occlusal structure, the patient expectations, and the clinician's experience should all be taken into consideration when choosing between these methods. Their long-term clinical performance should be the main focus of future research, especially with regard to the longevity of the restorations, their wear resistance, and marginal adaptation.

## 5. Conclusions

• Stamp and injection molding techniques offer promising solutions for direct composite restorations in young patients. Their ability to provide predictable, esthetic, and efficient restorations underscores their clinical significance.
• The stamp technique is best suited for Class I and II lesions, where occlusal anatomy is largely intact, whereas the injection molding technique is particularly beneficial for esthetic anterior restorations and select posterior restorations.
• Proper case selection, meticulous application, and periodic follow-ups remain essential to optimize their outcomes and ensure long-term success.
• Further clinical studies, such as randomized controlled trials with larger sample sizes, are essential to validate these findings and evaluate the long-term clinical performance of restorations.

## Data Availability

The data that support the findings of this study are available from the corresponding author upon reasonable request.
